# The Neural Bases of Event Monitoring across Domains: a Simultaneous ERP-fMRI Study

**DOI:** 10.3389/fnhum.2017.00376

**Published:** 2017-07-21

**Authors:** Vincenza Tarantino, Ilaria Mazzonetto, Silvia Formica, Francesco Causin, Antonino Vallesi

**Affiliations:** ^1^Department of Neuroscience, University of Padua Padua, Italy; ^2^Department of Information Engineering, University of Padua Padua, Italy; ^3^Neuroradiology Unit, Azienda Ospedaliera di Padua Padua, Italy; ^4^IRCCS San Camillo Hospital Foundation Venice, Italy

**Keywords:** EEG-fMRI, face processing, tool processing, sustained monitoring, transient monitoring, cognitive control

## Abstract

The ability to check and evaluate the environment over time with the aim to detect the occurrence of target stimuli is supported by sustained/tonic as well as transient/phasic control processes, which overall might be referred to as event monitoring. The neural underpinning of sustained attentional control processes involves a fronto-parietal network. However, it has not been well-defined yet whether this cortical circuit acts irrespective of the specific material to be monitored and whether this mediates sustained as well as transient monitoring processes. In the current study, the functional activity of brain during an event monitoring task was investigated and compared between two cognitive domains, whose processing is mediated by differently lateralized areas. Namely, participants were asked to monitor sequences of either faces (supported by right-hemisphere regions) or tools (left-hemisphere). In order to disentangle sustained from transient components of monitoring, a simultaneous EEG-fMRI technique was adopted within a block design. When contrasting monitoring versus control blocks, the conventional fMRI analysis revealed the sustained involvement of bilateral fronto-parietal regions, in both task domains. Event-related potentials (ERPs) showed a more positive amplitude over frontal sites in monitoring compared to control blocks, providing evidence of a transient monitoring component. The joint ERP-fMRI analysis showed that, in the case of face monitoring, this transient component relies on right-lateralized areas, including the inferior parietal lobule and the middle frontal gyrus. In the case of tools, no fronto-parietal areas correlated with the transient ERP activity, suggesting that in this domain phasic monitoring processes were masked by tonic ones. Overall, the present findings highlight the role of bilateral fronto-parietal regions in sustained monitoring, independently of the specific task requirements, and suggest that right-lateralized areas subtend transient monitoring processes, at least in some task contexts.

## Introduction

Flexible goal-directed behaviors require an adaptive cognitive control system that selects task-relevant contextual information and optimize its processing. A key cognitive control function supporting efficient systems is monitoring. This ability refers to a set of checking and evaluating processes, directed to assess stimuli or responses. As such, it represents a multifaceted function, recruited in a variety of apparently unrelated tasks and encompassing different sub-processes. For instance, in experiments on task performance monitoring, it denotes the ability to continuously check action outcomes in order to detect errors and adjust future action selection (Ridderinkhof and Ullsperger, 2004; Ullsperger et al., 2014). In time monitoring paradigms, it includes processes devoted to continuous updating of temporal/probability information in order to anticipate the occurrence of an upcoming stimulus and prepare motor response (Coull et al., 2000; Vallesi et al., 2007b, 2009; Coull, 2009). In prospective memory paradigms, it involves the active maintenance of task goals and the check of the environment to detect prospective targets (Guynn, 2003; Smith, 2003).

In all these tasks, monitoring is supported by both sustained/tonic and transient/phasic processes. When the occurrence of target stimuli/events is expected over time, sustained control processes should be instantiated, which promote and prepare individuals to their detection. In the same context, however, the occurrence of single stimuli elicits more transient control processes, since each of them should be assessed one-by-one. While the sustained monitoring processes are ubiquitous and span multiple trials, the transient monitoring processes operate within a trial. To give some examples: performance monitoring tasks requires the continuous assessment of whether ongoing actions match expected goals; on the other hand, it also involves more transient responses, such as error detection and subsequent action adjustments. Similarly, in time monitoring tasks, temporal expectations bias response preparatory processes over time. However, the actual onset of each target stimulus updates these expectations in a more trial-by-trial basis (Coull et al., 2016). In prospective memory tasks, individuals are engaged in the sustained maintenance of the prospective goal and, at the same time, they must check stimuli item-by-item in order to detect targets. This dual process is engaged especially in non-focal prospective tasks that require the detection of a stimulus feature unrelated to the ongoing task (McDaniel et al., 2015; Cona et al., 2016). The two qualitatively different cognitive controls processes might be assimilated to the proactive and reactive modes theorized by Braver (2012). They might act in a “semi-independent” manner, thus they may be both engaged simultaneously or one could be dominant on the other, in distinct moments in time, according to the experimental requirements (Gonthier et al., 2016).

Functional magnetic resonance imaging (fMRI) studies have provided evidence on distinct neural mechanisms subserving sustained and transient attentional control processes (Braver et al., 2003; Reynolds et al., 2009; McDaniel et al., 2013). These studies have used a mixed blocked/event design to disentangle between sustained and transient brain responses. By this methodological approach, block-related (sustained) functional brain activity and event-related (phasic) activity were extracted. Findings converged in showing a fronto-parietal network as the principal contributor of sustained attentional control processes. Specifically, in a prospective memory task, Reynolds et al. (2009) found that the activity of bilateral cortical regions of the middle frontal gyrus, namely the anterior (BA 10/46) and dorsolateral (BA 46/9) prefrontal cortex, regions of the superior and inferior parietal lobe (BA 7/40), and the anterior cingulate cortex were modeled as a sustained process, spanning the entire task block. Regions specifically engaged in item checking, in a transient (event-related) fashion, did not emerge. A selective transient response of the middle temporal gyrus was only found when the target was encountered. Similarly, McDaniel et al. (2013) found that bilateral areas, including the dorsolateral prefrontal cortex (middle frontal gyrus, BA 46), the anterior cingulate area (BA 32), the inferior frontal junction (precentral gyrus, BA 47/44), the frontal eye fields (precentral/middle frontal gyrus, BA 6), and superior parietal lobule (BA 7), were involved in sustained top-down attentional control. The transient brain response elicited by target trials involved the activity of some of the aforementioned fronto-parietal regions, namely, the left inferior frontal junction (BA 44), the frontal eye fields (BA 8/6) and the bilateral inferior parietal lobule (BA 40) plus the left anterior cingulate gyrus (BA 32), the bilateral anterior insula (BA 47) and ventral parietal cortex, which are likely involved in stimulus-driven processes. No evidence of transient processes related to non-target trials was provided. This is surprising, if one considering that, in a task requiring event monitoring, each stimulus (not just the targets) should undergo transient checking/evaluating processes (cf. Vallesi, 2014). Therefore, a question remains unexplored: are the transient item checking processes (i.e., the reactive control processes) obscured by more sustained monitoring processes because of the sluggish temporal resolution of fMRI?

To address this point, we designed an event monitoring experiment where participants were required to check series of visual stimuli over time for detecting the occurrence of target events (see also Poth et al., 2014). This event monitoring ability is crucial in many everyday activities, such as in the work of security officers who have to continuously inspect individuals as well as objects to identify critical targets and ensure the safe movement of a mass of people. In order to unveil the presence of sustained as well as transient monitoring processes, we conducted a simultaneous EEG-fMRI study. This multimodal technique is the gold-standard for characterizing spatial and temporal dynamics of brain activity over different time scales, within the same experimental session (Laufs, 2008; Mulert and Lemieux, 2009; Ullsperger and Debener, 2010; Huster et al., 2012; Jorge et al., 2014). In the current study, a blocked EEG-fMRI design was specifically devised. The co-registration approach allowed us, on the one hand, to capture the functional changes of brain regions associated with sustained activity across trials and inter-stimulus intervals (fMRI data) and, on the other hand, to capture the fast brain responses elicited by each event (ERP data). By doing so, we could disentangle tonic and phasic brain responses which are likely to at least partially overlap in space and time. By coupling block-related fMRI data and ERP data we were able to detect brain regions, among those showing a sustained activity, which actually intervened in a stimulus-driven fashion. Moreover, the simultaneous EEG-fMRI recording helped overcoming the limit of examining long-lasting and phasic components in separate blocks, as in previous studies. As such, this approach represents a possible alternative to mixed (Visscher et al., 2003; Petersen and Dubis, 2012) or “hybrid” (Braver et al., 2003) block/event-related fMRI designs.

Another missing point is whether there is a unique fronto-parietal circuit that mediates sustained monitoring processes, common to different task/material to be processed. Past research suggests that this possibility is very likely. However, direct evidence supporting this point is limited.

Along this line, a previous block-design fMRI experiment (Benn et al., 2014) represented an encouraging attempt to differentiate between long-lasting monitoring components and trial-by-trial ones and to compare monitoring processes involved in two different domains (numerical and visuo-spatial). The authors contrasted non-monitoring blocks to blocks that required monitoring either over time or trial-by-trial. The conjunction analysis showed that an extensive fronto-parietal network was associated with monitoring over time in both domains. This result suggested that domain-independent processes constituted the long-lasting component of monitoring. Compared to non-monitoring blocks, the trial-by-trial monitoring condition in the numerical domain activated the right superior parietal, left inferior parietal and bilateral superior and medial frontal gyri. Unfortunately, no evidence about the discrete, trial-by-trial monitoring component was collected in the visuo-spatial domain. Furthermore, areas activated in both sustained and transient monitoring were not investigated.

Recently, the domain-general nature of monitoring has been corroborated by an event-related potential (ERP) study. Here, participants had to monitor either verbal or spatial information while performing a concurrent verbal or spatial task, respectively (Capizzi et al., 2016). Stimuli in monitoring blocks elicited a more pronounced positivity over frontal and parietal scalp regions compared to stimuli in non-monitoring blocks, which was interpreted as reflecting greater attentional resources needs to maintain the focus of attention on the monitoring requirements.

To elucidate whether the same fronto-parietal network is recruited independently of the material to be monitored, in the current event monitoring experiment participants were asked to monitor the occurrence of different stimulus materials, within the same experimental session. To better dissociate the hemispheric contribution according to the domain, materials known to be processed by differently lateralized brain regions were used. Namely, in the control condition (i.e., non-monitoring blocks) participants performed a categorization task involving either faces or tools. While the processing of faces is usually subtended by dominant right temporal-occipital areas (e.g., Busigny et al., 2010; Frässle et al., 2016; for a recent review Yovel, 2016), the processing of tools mostly relies on a dominant left-lateralized fronto-parietal network (Grafton et al., 1997; Chao and Martin, 2000; Proverbio et al., 2013; Orban and Caruana, 2014; Perini et al., 2014). In monitoring blocks, they were also asked to monitor the occurrence of specific stimuli (i.e., faces or tools), which constituted the target stimuli. Since the probability of the target occurrence slightly varied across experimental blocks, participants had to continuously monitor stimuli over time in order to efficiently detect them.

Our primary hypothesis was that monitoring processes are mediated by a network of fronto-parietal cortical areas, with an important node in the lateral prefrontal cortex (Henson et al., 1999; Vallesi et al., 2007a,b, 2009; Shallice et al., 2008; Vallesi and Crescentini, 2011), and that these areas operate in a domain-independent fashion. By comparing blocks requiring monitoring to blocks not requiring this process, we expected that the activation of fronto-parietal areas would emerge, similarly for the two domains (i.e., faces and tools). The second hypothesis was that some of these fronto-parietal areas reflect sustained/tonic monitoring processes, whereas some others support more transient/phasic ones. By integrating block-related fMRI and ERP measures we expected to differentiate the neural bases of these two monitoring components.

## Materials and methods

### Participants

Twenty-two students from the University of Padua took part in the study. Data from two participants were discarded because of excessive head movements (>±3 mm in any translation direction) and two others because of low task performance (accuracy level <2.5 standard deviations). Therefore, the results are reported here for 18 participants (12 female; mean age: 23 years; age range: 20–28 years). They were all right-handed, as indicated by the Edinburgh Handedness Inventory (Oldfield, 1971; mean laterality score: 89.4, range: 70–100), and reported normal or corrected-to-normal visual acuity (MRI-compatible glasses were used when appropriate). The study was approved by the Bioethical Committee of the Azienda Ospedaliera di Padova and was conducted according to the guidelines of the Declaration of Helsinki. All participants signed a written informed consent prior to their participation and were paid 25 euro after the experiment.

### Stimuli

Stimuli consisted of pictures of faces and tools. The face pictures were downloaded from publicly available internet databases, after obtaining appropriate permissions when required (http://agingmind.utdallas.edu/download-stimuli/face-database/, Minear and Park, 2004; http://www.macbrain.org/resources.htm Tottenham et al., 2009; http://mmlab.ie.cuhk.edu.hk/archive/facesketch.html, Wang and Tang, 2009). All pictures were cropped and resized in ovals of 184 (width) × 272 (height) pixels. Hair and gender-related features (such as beard and make-up) were removed. Overall, a total of 50 face pictures were created, half female and half male. They belonged to different races, white/Caucasian, black/African-American, Hispanic, Middle-East, Indian. In addition, 20 pictures of Chinese individuals and 20 of older Caucasian adults were included as target stimuli to be monitored. Faces were selected that have a neutral expression and as few as gender-related features as possible.

The tool pictures were obtained via accurate selection on the web. Familiar tools (e.g., scissors, comb, guitar) were collected, that is, manipulable objects with a clear affordance, which implicitly suggests a motor interaction. The tool pictures were resized to maintain either the width or the height of the faces. Overall, a set of 50 tool pictures was selected, belonging to 15 unimanual and 15 bimanual categories. Twenty pictures of cooking tools (e.g., whisk, frying pan) and 20 of working tools (e.g., screwdriver, drill) were included as target stimuli to be monitored.

All pictures were converted in gray scale using the GIMP software (http://gimp.org). Luminance values were equalized across all images using the SHINE toolbox (Luminance Histogram Matching; Willenbockel et al., 2010), implemented in Matlab. Overall, a total of 280 face pictures and 280 tool pictures were presented across the whole experiment.

A pilot study was carried out on 12 subjects to ensure that all stimuli were easily recognizable and correctly categorized as female/male faces and unimanual/bimanual objects with at least 90% of accuracy. In addition, a set of 40 scrambled images (272 × 272 pixel) were created by averaging the pixels of 20 randomly selected pictures and were used during rest/fixation phases. All images were presented centrally on a 64 (width) × 40 (height) cm screen (InVivo Esys Display, Gainesville, FL, USA), 1,280 × 800 pixel resolution, on a white background. Faces were 9 (width) × 14 (height) cm of size and objects were contained in a 14 (width) × 14 (height) cm square. The screen was placed at the head of the bore and the images were visible to the participants through a double mirror system mounted on the head coil, with the head of participants lying 150 cm from the monitor.

### Procedure and task

**Figure 1:**
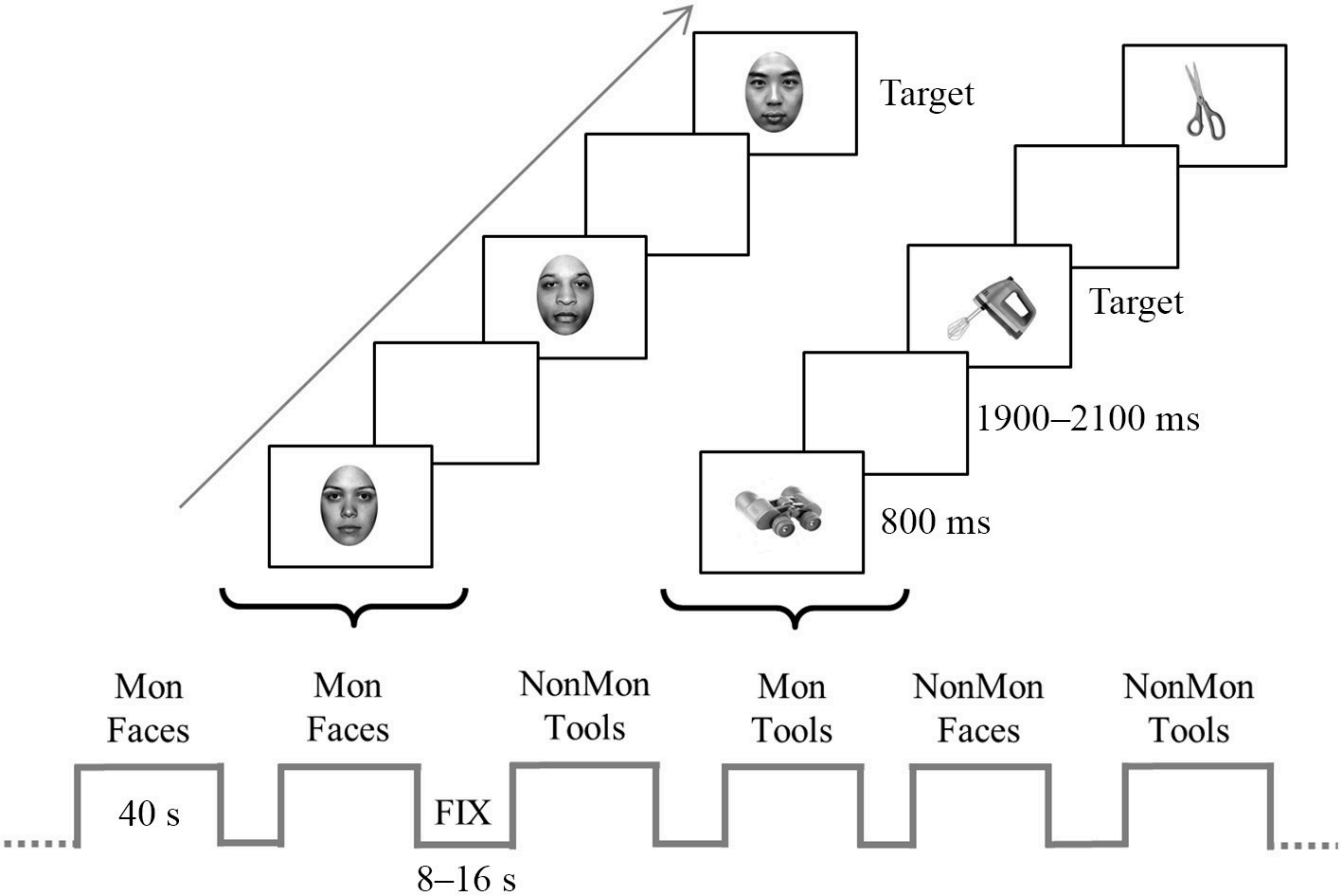
Schematic representation of the experiment. The bottom of the figure displays an example of block sequence; blocks (40-s) were alternated with fixation blocks (8–16 s). The top of the figure depicts an example of trial sequence within a two monitoring (Mon) blocks. Faces and tools were displayed for 800 ms in each trial and appeared in separate blocks. In the figure, the target stimulus in the Face Mon block is represented by a Chinese face, whereas in the Tool Mon block is the picture of a blender. In the non-monitoring (NonMon) blocks the subject has to categorize the picture as female/male face or unimanual/bimanual tool. In the Mon blocks the subjects has to perform the categorization task and to detect to the target stimuli.

An illustrative picture of the experimental procedure is reported in Figure 1. Four types of blocks were pseudo-randomly presented, namely monitoring blocks and non-monitoring blocks containing faces (hereafter named “Mon Faces” and “NonMon Faces,” respectively), monitoring blocks and non-monitoring blocks containing tools (hereafter named “Mon Tools” and “NonMon Tools,” respectively). The entire experiment contained 10 blocks of each type (Mon Faces, NonMon Faces, Mon Tools, and NonMon Tools), which were grouped in 5 scanning runs of 8 blocks each (2 for each type). Each run consisted of alternating cycles of fixation (A) and task (B) blocks presented in an ABAB succession. Fixation blocks (denoted by a centrally presented scrambled image) lasted randomly 8, 10, 12, 14, or 16 s (mean duration 12 s); task blocks were 40 s long and included 14 trials, containing 7 female and 7 male faces, or 7 unimanual and 7 bimanual tools. Monitoring blocks included from 2 to 6 target stimuli. Each stimulus was presented centrally for 800 ms, followed by a blank with an inter-stimulus interval continuously varying between 1,900 and 2,100 ms. This small random variation of the inter-trial interval ensured that the stimuli were not locked to multiples of the slice acquisition frequency, minimizing the influence of any residual MRI artifact in the EEG trace. Stimulus presentation and response collection were controlled by Eprime 2 software (Schneider et al., 2002).

In Face blocks participants had to decide whether the face gender was female or male. In Tool blocks they were asked to decide whether the object is generally manipulated with one hand (unimanual objects) or with both hands (bimanual objects). Responses were given by pressing one of two buttons with the two index fingers lying on two-button MRI-compatible response boxes. All possible combinations between category (female/male, unimanual/biamanual) and responding hand were counterbalanced across participants. In addition to this ongoing task, in half of the experimental blocks participants were asked to perform a monitoring task, namely they had to detect specific categories of faces or tools (targets). In Mon Faces blocks they were asked to detect either Chinese faces or faces of people older than 50 years; in Mon Tool blocks they were asked to detect either cooking tools or working utensils (in Italian: “attrezzi da lavoro”^1^). The two target categories never appeared together in the same block. Each block was preceded by an instruction screen (2 s in the fixation block, 6.5 s in the task blocks) that indicated the task to be executed, the target category to be monitored, and reminded the stimulus-response mappings. In order to verify that participants correctly detected the target stimuli, at the end of Mon blocks they were asked to estimate the approximate number of targets. The numbers from 1 to 6 appeared, two at a time, and participants had to press the response button (beneath the left or right index) corresponding to the number of targets that they had estimated. Participants were instructed not to count but to focus on target detection. Furthermore, they were informed that the Monitoring blocks had a 100% probability to contain at least 1 target stimulus in order to encourage the involvement of monitoring processes.

A practice session was performed the day before, outside the scanner. During this session one sample of each type of block was presented and trial-by-trial feedbacks on participants' accuracy were provided. If participant's overall accuracy was below 80%, the practice was repeated until she/he reached this criterion. The experimenter clarified any doubt on the experimental session and ensured that the instructions were clearly understood.

### fMRI signal acquisition and preprocessing

MR images were acquired using a 3T Ingenia Philips whole-body scanner (Philips Medical Systems, Best, The Netherlands) equipped with a 32-channel head-coil, at the Neuroradiology Unit of the Azienda Ospedaliera of Padova. Functional volumes were obtained using a whole head T2^*^-weighted echo-planar image (EPI) sequences (repetition time, TR: 2,000 ms; echo time, TE: 35 ms; 25 axial slices with ascending acquisition; voxel size: 2.4 × 2.4 × 4.8 mm; flip angle, FA: 90°; field of view, FOV: 230 mm, acquisition matrix: 84 × 80; SENSE factor: 2 in anterior-posterior direction).

Special care was taken to ensure that frontal areas and cerebellum would be included in the imaging volume. Small foam cushions were placed around the participant's head to minimize head movements and to ensure a comfortable position; they also wore earplugs to reduce acoustic noise. A total of 282 EPI images were acquired for each of the five runs. Two dummy scans at the beginning of each run were not acquired. High-resolution T1-weighted anatomical images (TR/TE: 8.1/3.7; 180 sagittal slices; FA: 8°; voxel size: 0.49 × 0.49 × 1 mm; FOV: 220 mm; acquisition matrix: 220 × 220) were acquired after the functional runs.

The fMRI data pre-processing and statistical analyses were performed using SPM12 (Statistical Parametric Mapping software; Wellcome Department of Cognitive Neurology, London, UK; http://www.fil.ion.ucl.ac.uk/spm). Functional images were spatially realigned to compensate for participants' head movements during the experiment using a 4th degree B-Spline interpolation and a mean image of the realigned volumes was created. For normalization, the 6-parameter rigid-body transformation from the mean image to the anatomical image was concatenated with a transformation from the anatomical image to the standard Montreal Neurological Institute (MNI) template (2 mm^3^ voxel-size) and applied to all volumes. Normalization to MNI was performed by combining registration and tissue classification into a single generative model, which also includes parameters that account for image intensity non-uniformity. The functional images were then spatially smoothed with an 8 mm full-width-at-half-maximum Gaussian filter.

### EEG signal acquisition and preprocessing

The EEG signal was recorded using a MR-compatible system (Brain Products, Munich, Germany), connected to 64 sintered Ag/AgCl ring electrodes, equipped with current-limiting 5-kΩ resistors and mounted on an elastic cap (BrainCap MR) according to the extended 10–20 system. Reference and ground channels were located over FCz and AFz, respectively. An electrode placed in the middle of participants' back, approximately 4 cm left to the spine, was used to acquire the electrocardiographic (ECG) signal. Impedance values were kept below 5 kΩ. Raw data were band-pass filtered between 0.016 and 250 Hz digitized at a sampling rate of 5 kHz. The EEG was monitored while scanning using online correction software (RecView 1.4, Brain Products). Overall, the EEG recording procedure was performed according to safety and data quality guidelines provided by Mullinger et al. (2013).

The EEG data preprocessing was performed using either BrainVision Analyzer 2.1 (Brain Products) or EEGLAB 12.0 (Delorme and Makeig, 2004), according to each specific preprocessing step. The gradient artifact (GA) was removed from EEG data using the fMRI artifact slice template removal (FASTR) algorithm (Niazy et al., 2005), implemented in EEGLAB (FMRIB plug-in). A total of 31 consecutive volume artifacts were included in the averaging window for computing the artifact template. The gradient residual artifacts were removed by the Optimal Basis Set (OBS) procedure (Niazy et al., 2005). The resulting EEG signal was low-pass filtered by applying a windowed sinc FIR filter, with a cut-off frequency of 40 Hz, a Kaiser Window type with a beta of 5.65, a maximum pass-band deviation of 0.001 and a transition band of 10 Hz (Widmann et al., 2014). The ballistocardiographic (BCG) artifact was removed using a semi-automatic procedure implemented in BrainVision Analyzer. As an initial step, the R peaks of every heart pulse were automatically detected and marked on the ECG channel. Visual inspection was then conducted on the whole ECG signal to ensure the correct positioning of all the peaks. Finally, the artifact was removed from all EEG channels by means of an average template subtraction, analogously to the procedure implemented for the GA (Allen et al., 1998, 2000; Niazy et al., 2005).

The EEG signal was then down-sampled to 500 Hz. The BCG artifact residuals, ocular movements and muscle artifacts were removed by means of an Independent Component Analysis (ICA; Debener et al., 2007; Mantini et al., 2007), based on an extended Infomax algorithm (Bell and Sejnowski, 1995). The continuous EEG signal was segmented in epochs ranging from -200 ms before stimulus onset to 1,000 ms after stimulus presentation. The resulting epochs were then baseline-corrected using a time window from −200 to 0 ms. Data were re-referenced to the average of all electrodes, with the exception of the ECG channel. To allow a reliable integration with fMRI block analysis, all trials belonging to a block were included in the ERP average. Afterwards, the resulting block-by-block ERPs were averaged for each condition, namely NonMon Faces, Mon Faces, NonMon Tools, Mon Tools.

## Data analysis

### Behavioral data analysis

Accuracy and response times (RTs) on the female/male and unimanual/bimanual tasks were examined in order to investigate the effect of Monitoring (NonMon, Mon) and Domain (Faces, Tools). Due to the non-normal distribution of accuracy data, the non-parametric Friedman's ANOVA was performed. Post hoc Wilcoxon signed-rank tests were then run on pairs of conditions. A 2 × 2 repeated measure ANOVA model was used to test the effect of Monitoring and Domain factors on mean RTs. For all the behavioral analyses, the significance level was set at α = 0.05 and corrected for multiple comparisons in post hoc tests using the Bonferroni procedure. The partial eta squared (η^2^) was computed to quantify the effect size. Statistical analyses were conducted using the SPSS 22 software (IBM).

### fMRI data analysis

For each participant, first-level analyses were performed using a General Linear Model (GLM). Five task-related regressors entered the GLM, one for each block type (Mon Tools, NonMon Tools, Mon Faces, NonMon Faces and Fixation), which were convolved with a canonical Hemodynamic Response Function (HRF). Six additional regressors derived from the motion correction step were also included in the design matrix as regressors of no-interest, to account for variance associated with head movements. Slow signal drifts were removed using a 240 s high-pass filter. The hemodynamic response for each of the four experimental conditions was contrasted with the average of the hemodynamic response in the Fixation blocks, used as baseline. The second-level (i.e., group) SPM maps were generated from the individual contrast maps using a random-effect model. Namely, a 2 (Domain: Faces, Tools) × 2 (Monitoring: Mon, NonMon) full-factorial ANOVA was performed and the following specific t-contrasts were computed: Faces > Tools and Tools > Face (collapsed for Monitoring factor), Mon Faces > NonMon Faces and Mon Tools > NonMon Tools (collapsed for Domain factor).

Moreover, a conjunction analysis was performed to investigate those voxels which were commonly activated in Mon compared to NonMon in Face and Tool blocks. The whole brain was considered in the analysis. The statistical significance of the blood oxygenation level-dependent (BOLD) response changes was set at *p* < 0.05 using a voxel-level family-wise error (FWE) correction for multiple comparisons. A cluster-size threshold of 20 contiguous voxels was further applied (Lieberman and Cunningham, 2009). The anatomical regions corresponding to MNI coordinates of the peak voxels within each cluster were extracted by referring to the probabilistic Anatomical Automated Labeling (AAL) atlas, implemented in SPM12 (Tzourio-Mazoyer et al., 2002, http://www.gin.cnrs.fr/AAL2). The “whereami” toolbox of AFNI (Cox, 2012) was used to find the likely Brodmann area (BA) for each cluster.

### ERP data analysis

The mean global field power (GFP) was computed for each subject and condition. This measure summarizes the contribution of all electrodes point-by-point and indexes global modulations in the strength of the electric field (Lehmann and Skrandies, 1980). Mathematically, it equals the root mean square of the average-referenced amplitude values across all electrodes at a given point in time. The extraction of ERP components was time-centered on the interval where differences between conditions in GFP emerged based on paired *t*-tests (Figure S1). Analogously, the electrodes to be considered for each ERP component were determined by examining the topographical distribution of *t*-tests (t-maps), which resulted from contrasting individual ERP averages between pairs of conditions. The ERP components were quantified in terms of peak amplitude, peak latency or mean amplitude. The effects of Monitoring and Domain factors were assessed by means of 2 × 2 repeated-measure ANOVAs.

### EEG-fMRI integration

The relationship between the ERP and the fMRI responses was investigated using the integration-by-prediction method (Debener et al., 2006; Mulert et al., 2008; Eichele et al., 2009). This method, which was developed for event-related designs, in this study was adapted to a block design. First of all, all five runs were concatenated. To build the correct concatenation model, the high-pass filter and temporal non-sphericity calculations were corrected to account for the original run lengths. The ERP components that were found to be associated with the monitoring process in the conventional analyses were considered for the integration analysis. As it will be detailed in the result section, for each block the mean voltage amplitude either of the frontal or the parietal ERP components was extracted and entered into two separate GLMs as parametric modulators of the BOLD response. For example, the mean ERP amplitude extracted from a Mon Face block over frontal electrode sites modulated the estimated BOLD response in that block.

Overall, 4 ERP regressors were included in the first-level GLM, together with the 11 conventional regressors (4 for experimental conditions, 1 for fixation, and 6 for movement parameters). Before entering the model, each ERP regressor was orthogonalized with respect to its conventional regressor by mean centering it. This procedure allows the detection of hemodynamic responses specifically related to variations in the ERP response and not to some general feature of the task experimental condition (Debener et al., 2006; Mulert et al., 2008; Eichele et al., 2009). Afterwards, the ERP regressors were convolved with the canonical HRF function. At the group level, the relationship between ERP amplitudes and BOLD responses was assessed using a 2 × 2 full-factorial ANOVA model. The t-contrasts between the ERP-related regressors were generated. Namely, the activations obtained from the Mon Faces > NonMon Faces and Mon Tools > NonMon Tools contrasts were used as inclusive masks, in order to identify the brain regions associated with the ERP components within the clusters of voxels emerged from the conventional fMRI analysis.

## Results

### Behavioral results

Both data on accuracy and RTs confirmed the presence of a Monitoring cost (lower performance in Mon compared to NonMon blocks). Mean values are summarized in Table 1. The Friedman's test showed significant differences in accuracy on the categorization task across conditions [χ^2^_(3, 18)_ = 36.27, *p* < 0.001). Post hoc Wilcoxon's tests on pairs of variables revealed that the accuracy in Mon blocks was lower than accuracy in NonMon blocks, for both Faces (*Z* = 3.59, *p* < 0.001) and Tools (*Z* = 3.74, *p* < 0.001). No differences in accuracy emerged between Faces and Tools (both *Zs* < 1.34, *ps* > 0.178). The 2 (Faces, Tools) × 2 (MonNon, Mon) ANOVA performed on mean RTs yielded a significant main effect of Monitoring [*F*_(1, 17)_ = 60.57, *p* < 0.001, η^2^ = 0.781] and a significant Monitoring × Domain interaction [*F*_(1, 17)_ = 11.41, *p* = 0.004, η^2^ = 0.402]. The interaction revealed that while the Monitoring cost was present in both domains, it was higher in Tools compared to Faces [*t*_(17)_ = 3.38, *p* < 0.004]. The mean accuracy in the estimation of the number of targets reported at the end of each monitoring block was 92.7% (SD = 21.5) for Faces and 98.3% (SD = 3.8) for Tools (*Z* = 1.00, *p* = 0.317).

**Table 1:** Mean response times (RT) and percentage of accurate responses (ACC) on the Non-Monitoring (NonMon) and Monitoring (Mon) blocks by Stimulus material (Face, Tools).

	**Faces**		**Tools**	
	**ACC (%)**	**RT (ms)**	**ACC (%)**	**RT (ms)**
NonMon	97.3 (3.9)	583 (94)	98.6 (1.0)	573 (64)
Mon	93.8 (3.5)	643 (112)	93.1 (2.9)	658 (102)

*Standard deviation values are reported in parentheses*.

### fMRI results

**Figure 2:**
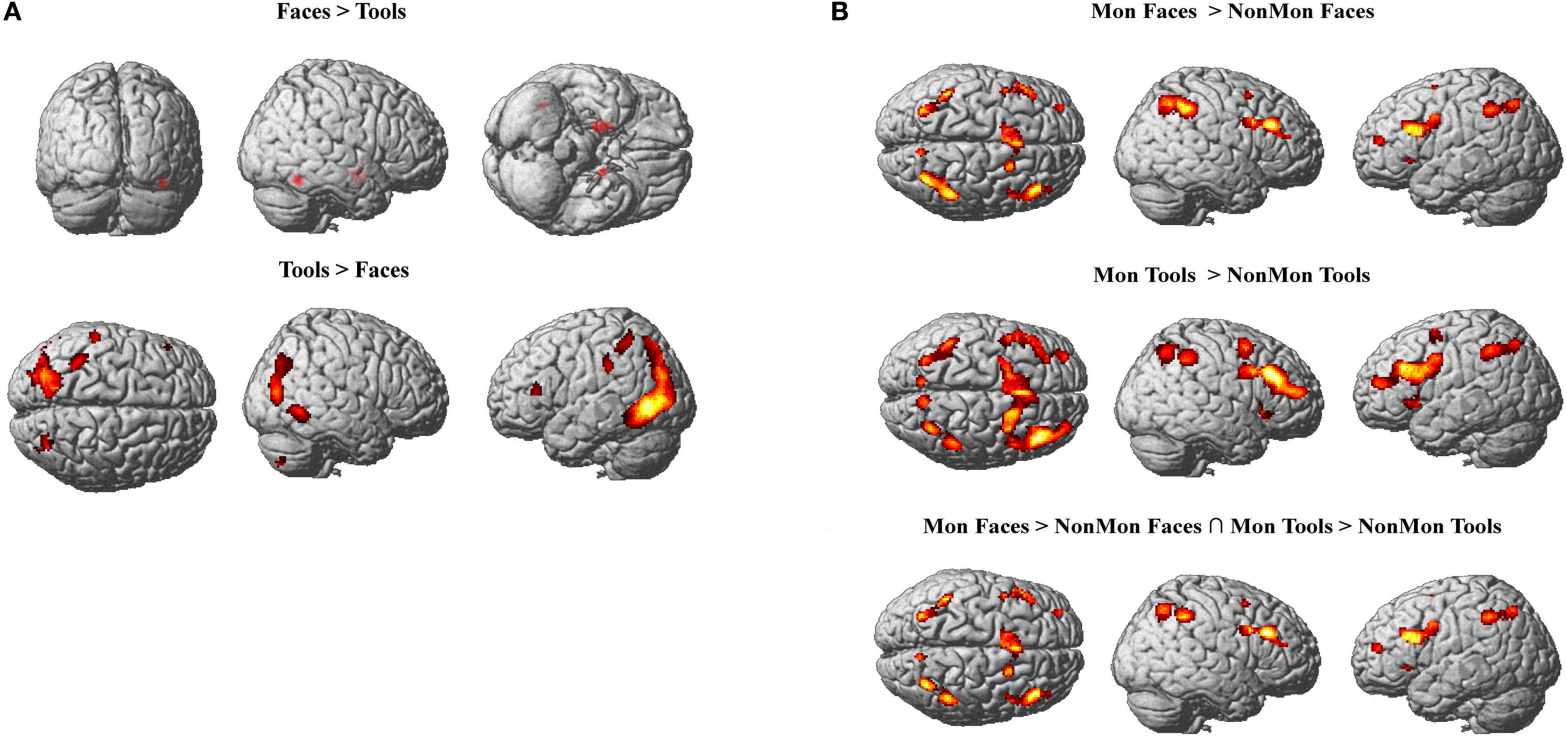
**(A)** Active clusters in Face blocks relative to Tool blocks (top) and in Tool blocks relative to Face blocks (bottom). **(B)** Active clusters in Monitoring relative to Non-Monitoring blocks in the Face domain (top), in Tool domain (middle) and in both domains (bottom). All displayed activations emerged at *p* < 0.05 (voxel-level FWE correction) and *k* > 20.

In Tables 2, 3 the results of the 2 × 2 full-factorial ANOVA (*p* < 0.05, voxel-level FWE correction) are reported. Specifically, the Table 2 contains the between Faces and Tools t-contrasts. Compared to the Tools, the Faces yielded a positive activation of the right fusiform gyrus and of the amygdala, bilaterally (Figure 2A, top panel). Compared to the Faces, the Tools processing activated a broader set of brain regions encompassing the fusiform gyrus bilaterally (mainly in the left hemisphere), the middle temporal gyrus and the middle occipital gyrus bilaterally, the left inferior parietal lobule (IPL), comprising the supramarginal gyrus and the intra-parietal sulcus (IPS), the right inferior temporal gyrus, the left inferior frontal gyrus (pars triangularis), and the right cerebellum (Figure 2A, bottom panel).

**Table 2:** Brain regions showing significant fMRI activations (voxel-level *P*_FWE_ < 0.05) in the Faces vs. Tools blocks.

**Anatomical region**	**Side**	**BA**	**Cluster size**	**MNI coordinates (mm)**			***T*-values**
				**x**	**y**	**z**	
**Faces > Tools**							
Amygdala	R		187	22	−6	−14	7.98
Fusiform gyrus	R	37	58	38	−56	−18	7.77
Amygdala	L		38	−18	−6	−18	5.42
**Tools > Faces**							
Fusiform gyrus	R	37	1,103	32	−46	−8	15.28
Fusiform gyrus	L	37	5,260	−30	−46	−12	14.83
Middle temporal gyrus	L	37		−48	−62	−4	14.36
Middle occipital gyrus	L	19		−42	−80	8	12.12
Middle occipital gyrus	R	19	816	30	−72	34	8.62
Middle occipital gyrus	R	39		42	−78	10	8.13
Inferior temporal gyrus	R	37	202	50	−58	−6	8.61
Supramarginal gyrus	L	40	93	−58	−32	34	6.67
Inferior parietal lobule	L	40		−54	−32	44	5.01
Inferior frontal, triangular part	L	45	61	−52	30	12	6.55
Cerebellum	R		46	28	−76	−48	6.49
Inferior parietal lobule	L	40	192	−36	−50	56	6.14
Inferior parietal lobule	L	40		−38	−42	48	5.75

*L and R stand for left and right hemisphere, respectively. MNI coordinates and t-values are reported for all peaks observed within a cluster. Anatomical labels derived from the Anatomical Automated Labeling (AAL) atlas*.

**Table 3:** Brain regions showing significant fMRI activation (voxel-level *P*_FWE_ < 0.05, unless otherwise specified) in the Monitoring vs. Non-Monitoring blocks.

**Anatomical region**	**Side**	**BA**	**Cluster size**	**MNI coordinates (mm)**			***T*-values**
				**x**	**y**	**z**	
**Faces: Mon > NonMon**							
Superior parietal lobule	L	7	519	−28	−64	46	8.83
Inferior parietal lobule	L	40		−40	−48	40	6.80
Inferior parietal lobule	L	40		−46	−46	50	6.08
Supplementary Motor Area	L	6	589	−6	10	52	8.79
Superior frontal gyrus, dorsolateral	R	6		20	10	54	5.92
Supplementary Motor Area	L	6		−16	2	66	5.40
Angular gyrus	R	7	860	34	−62	48	7.90
Inferior parietal lobule	R	40		44	−46	42	7.12
Inferior parietal lobule	R	40		36	−52	44	6.72
Inferior frontal gyrus, opercular part	R	9/44	576	40	10	30	7.27
Middle frontal gyrus	R	9		42	30	32	7.01
Middle frontal gyrus	R	46		40	34	22	5.40
Inferior frontal gyrus, triangular part	L	44/45	658	−40	20	26	6.87
Inferior frontal gyrus, triangular part	L	45		−50	24	28	6.69
Precentral gyrus	L	9		−44	2	36	6.23
Insula	L	47	85	−32	26	0	6.57
Middle frontal gyrus	L	10	56	−30	50	16	6.19
Precuneus	R	7	84	8	−66	46	6.07
Insula	R	47	24	32	26	−4	5.46
**Tools: Mon > NonMon**							
Supplementary Motor Area	L	6	3,707	−6	10	52	10.26
Middle frontal gyrus	R	9		42	30	32	9.41
Superior frontal gyrus, dorsolateral	R	6.32		20	8	52	7.62
Inferior frontal gyrus, triangular part	L	44/45	2,485	−36	22	26	10.01
Insula	L	47		−32	26	0	8.49
Precentral gyrus	L	9		−44	4	30	7.84
Superior parietal lobule	L	7	772	−28	−64	48	7.98
Inferior parietal lobule	L	40		−42	−46	42	7.80
Insula	R	47	242	32	26	−4	7.83
Precuneus	R	7	198	10	−66	48	7.23
Angular	R	7	520	34	−62	48	7.11
Inferior parietal lobule	R	40		50	−40	48	6.06
Inferior parietal lobule	R	47		44	−46	44	5.95
Precuneus	L	7	75	−10	−70	52	6.60
Middle cingulate gyrus	R	23	39	6	−22	26	6.22
**Mon > NonMon (Conjuction)**							
Supplementary Motor Area	L	6	577	−6	10	52	8.79
Superior frontal gyrus, dorsolateral	R	6		20	10	54	5.92
Supplementary Motor Area	L	6		−16	2	64	5.35
Superior parietal lobule	L	7	486	−28	−64	48	7.98
Inferior parietal lobule	L	40		−40	−48	40	6.80
Angular gyrus	R	7	436	34	−62	48	7.11
Inferior parietal lobule	R	40		48	−40	46	6.01
Inferior frontal gyrus, opercular part	R	9	524	42	10	32	7.02
Middle frontal gyrus	R	9		42	30	32	7.01
Middle frontal gyrus	R	46		40	34	22	5.40
Inferior frontal gyrus, triangular part	L	44	652	−40	20	26	6.87
Inferior frontal gyrus, triangular part	L	45		−50	24	28	6.69
Precentral gyrus	L	6		−44	2	36	6.23
Insula	L	47	85	−32	26	0	6.57
Middle frontal gyrus	L	10	56	−30	50	16	6.19
Precuneus	R	7	84	8	−66	46	6.07
Insula	R	47	24	32	26	−4	5.46
Mon Faces > NonMon Faces–ERP modulation^*^							
Inferior parietal lobule	R	40	63	46	−50	42	3.35
Middle frontal gyrus	R	9	85	48	24	34	3.31
Middle frontal gyrus	R	9		38	26	32	2.61

*L and R stand for left and right hemisphere, respectively. MNI coordinates and t-values are reported for all peaks observed within a cluster. Anatomical labels derived from the Anatomical Automated Labeling (AAL) atlas*.

* *Voxel-level uncorrected p < 0.005, k > 52*.

The contrast between Mon and NonMon blocks showed the recruitment of extensive clusters of voxels including bilateral frontal and parietal cortical regions, consistently associated with sustained control processes in previous studies (Table 3 and Figure 2B). The activated regions were very similar between the two domains. The conjunction analysis revealed the areas commonly involved in both domains (Table 3 and Figure 2B, bottom panel). Specifically, in the frontal right hemisphere, the inferior portions of the middle frontal gyrus (MFG), the inferior frontal gyrus (IFG, pars opercularis), and the dorsolateral portion of the superior frontal gyrus (SFG) were activated. At the parietal level, the angular gyrus, portions of the IPL, comprising the supramarginal gyrus, and a smaller cluster in the right precuneus were found. In the left hemisphere, the activity mainly included more ventral PFC regions (i.e., the pars triangularis of the IFG), and a small cluster peaking in a frontopolar portion of the MFG. Moreover, a medial portion of the SFG corresponding to the supplementary motor area (pre-SMA) and extending to anterior cingulate cortex (ACC), and a portion of the precentral gyrus corresponding to the inferior frontal junction (IFJ, i.e., the intersection between the precentral sulcus and the inferior frontal sulcus) were also active in Mon blocks. At the parietal level, the activation clusters in the left hemisphere comprised portions of the superior parietal lobule (SPL) and IPL. Both the right and the left parietal clusters contained the IPS. Smaller clusters involved the insula bilaterally.

Differential patterns of activations for Mon vs. NonMon blocks between Face and Tools were explored through second level interaction t-contrasts (weights for the Mon Face, NonMon Face, Mon Tools, NonMon Tools blocks: 1 −1 −1 1 and −1 1 1 −1). This analysis revealed that no regions exhibited differential monitoring-related activations between the two domains.

### ERP results

**Figure 3:**
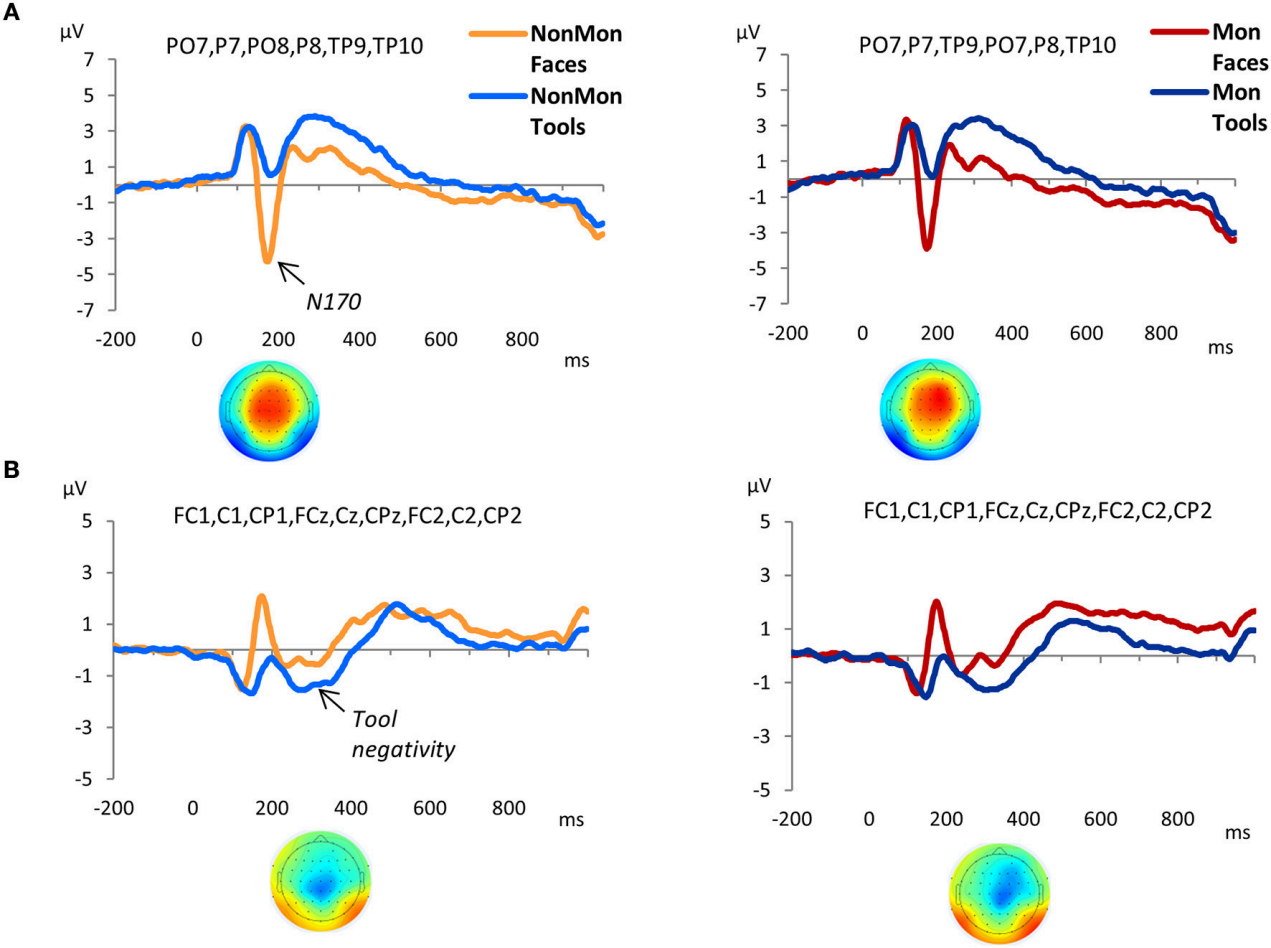
Grand-average waveforms of stimulus-locked ERPs for each Domain (Faces vs. Tools), separated by Monitoring condition (Non-Monitoring vs. Monitoring). **(A)** face-sensitive ERP component, N170; **(B)** tool-sensitive ERP component, here named Tool negativity. The zero time point corresponds to the onset of the stimulus. The t-maps in panel A represent the topographical distribution of *t*-tests values obtained from the difference between ERP averages in Face and Tool blocks from 140 to 210 ms **(A)**, and from the difference between ERP averages in Tool and Face blocks from 240 to 340 ms **(B)**. *T*-test values range from −10 to +10.

As expected, a large negative peak was evoked at the onset of the pictures of faces over parieto-occipital and temporo-occipital electrodes (Figure 3A). This peak clearly represents a N170 component. The amplitude of the peak was contrasted across experimental conditions. Based on previous literature (Itier and Taylor, 2004; Rossion and Jacques, 2008), and on temporal and spatial information derived from the GFP and the t-maps (Figure S1), the latency of the maximum negative peak was extracted from 140 to 210 ms in PO7, P7, PO8, P8, TP9, TP10 electrodes. The amplitude mean over a 12 ms time-window around the identified peak latency was measured. The 2 (NonMon, Mon) × 2 (Faces, Tools) ANOVA yielded a significant main effect of Domain [*F*_(1, 17)_ = 74.85, *p* < 0.001, η^2^ = 0.815], which statistically confirmed the presence of a larger negative peak for Faces. Moreover, a Monitoring × Domain interaction emerged [*F*_(1, 17)_ = 6.96, *p* = 0.017, η^2^ = .815] that revealed a decrease of the N170 amplitude in Mon blocks compared to NonMon blocks only in the Face domain (*p* = 0.039). The mean latency of the peak was 177 ms (SD = 10) and 178 ms (*SD* = 10) for Faces in the NonMon and Mon blocks respectively, and 181 (SD = 9) and 184 (SD = 10) for Tools. The ANOVA on mean latencies showed a main effect of Domain [*F*_(1, 17)_ = 7.71, *p* = 0.013, η^2^ = 0.312], which confirmed that the peak emerged slightly earlier for Faces.

The GFP exhibited an increased strength of the electric field in the Tool blocks compared to Face blocks from 240 to 340 ms (Figure S1). The t-maps revealed that this modulation mainly affected the following electrodes: FC1, FCz, FC2, C1, Cz, C2, CP1, CPz, and CP2. The waveforms of picture-evoked potentials plotted over these electrodes showed a negative deflection that characterized the responses to tools compared to faces (Figure 3B). Therefore we named this ERP component as “tool negativity.” The mean ERP amplitude of this component was extracted for each subject and condition in these electrodes, from 240 to 340 ms, and submitted to a 2 × 2 ANOVA. This analysis yielded a significant main effect of Domain [*F*_(1, 17)_ = 38.80, *p* < 0.001, η^2^ = 0.695] which confirmed larger negative ERP responses for Tools. Moreover, a main effect of Monitoring [*F*_(1, 17)_ = 7.58, *p* = 0.014, η^2^ = 0.308] revealed more positive ERP waveforms in Mon blocks.

**Figure 4:**
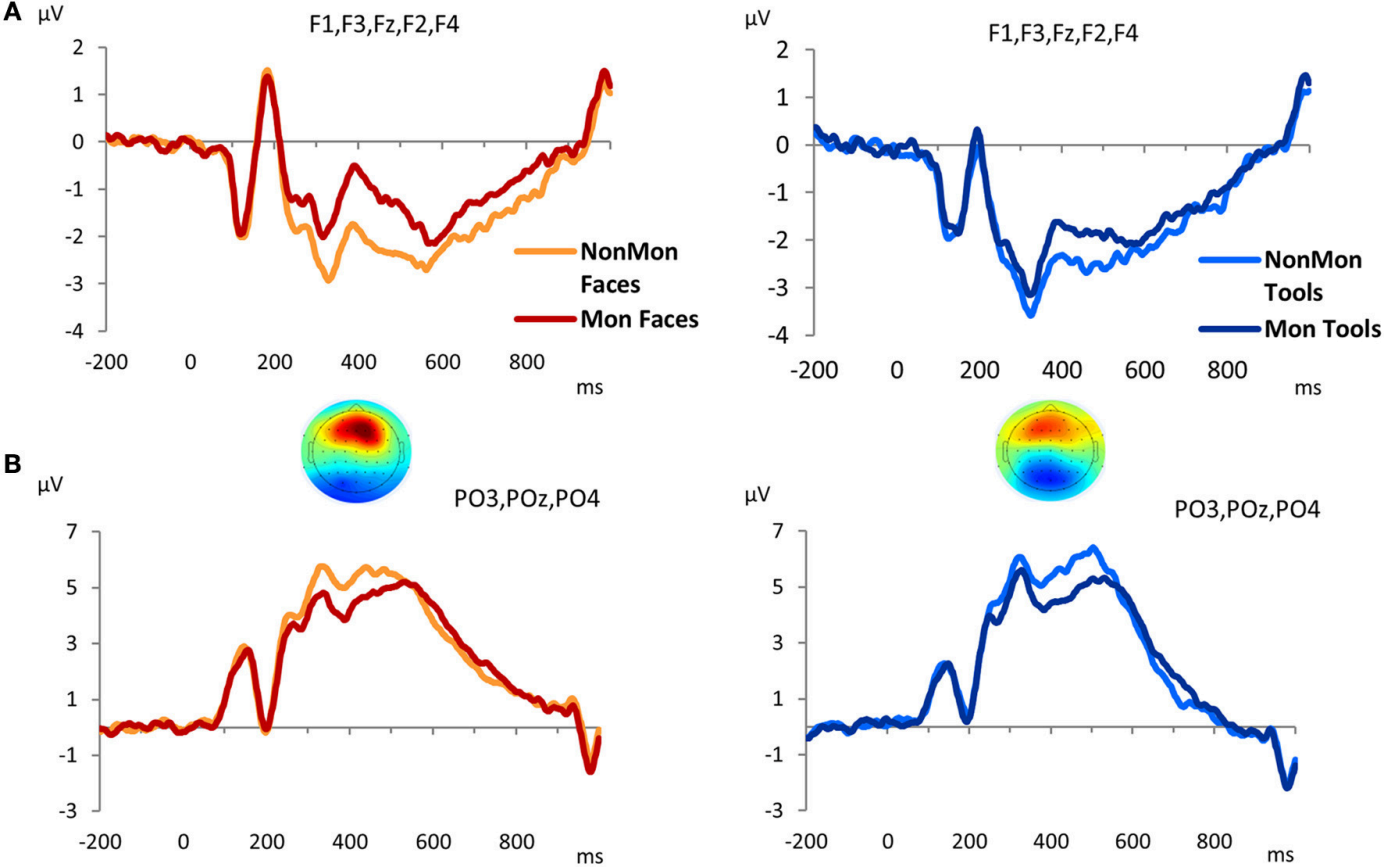
Grand-average waveforms of stimulus-locked ERPs for each Monitoring condition (Non-Monitoring vs. Monitoring), separated by Domain (Faces vs. Tools), over frontal **(A)** and parietal **(B)** electrodes. The zero time point corresponds to the onset of the picture. The t-maps represent the topographical distribution of *t*-tests in the 320–520 ms time window. *T*-test values range from −6 to +6.

The GFP revealed that differences between Mon and NonMon blocks emerged from 320 to 520 ms over frontal as well as parietal sites in both domains, as confirmed by the t-maps (Figure S1). The frontal ERP component was characterized by a positive deflection in the Mon blocks compared to NonMon blocks (Figure 4A), whereas the parietal component was characterized by a negative deflection in the Mon blocks (Figure 4B). Given its spatio-temporal trend, the latter component might be assimilated to a P3. Based on the t-maps, the frontal component was quantified in terms of mean ERP amplitude over F3, F1, Fz, F2, and F4 electrodes, whereas the parietal component was quantified in terms of mean ERP amplitude over PO3, POz, and PO4 electrodes.

To sum up, two monitoring-related ERP components emerged, a frontal positivity and a parietal negativity. The 2 × 2 ANOVA on the frontal ERP component showed a main effect of Monitoring [*F*_(1, 17)_ = 48.18, *p* < 0.001, η^2^ = 0.739] that revealed that the pictures evoked more positive ERPs over frontal electrode sites in Mon compared to NonMon blocks. In addition, a main effect of Domain emerged [*F*(1, 17) = 8.20, *p* = 0.011, η^2^ = 0.325] that showed that the ERPs (P3) were more positive for Faces compared to Tools. No significant interaction was found [*F*_(1, 17)_ = 2.66, *p* = 0.121, η^2^ = 0.136]. On the other hand, the analysis of ERP amplitude over parietal sites yielded a significant effect of Monitoring [*F*_(1, 17)_ = 51.19, *p* < 0.001, η^2^ = 0.751] that revealed an amplitude decrease in Mon compared to NonMon blocks. Neither the main effect of Domain [*F*_(1, 17)_ = 0.179, *p* = 0.678, η^2^ = 0.01] nor the Monitoring × Domain interaction [*F*_(1, 17)_ = 0.478, *p* = 0.498, η^2^ = 0.027] were significant.

In all the above reported analyses, both fMRI and ERP, Target and Non-Target trials in the Mon blocks were collapsed. In order to examine the presence of differences in the event-related brain activity between Non-Target and Target trials within the Mon blocks compared to the NonMon trials, we analyzed the ERP amplitude in the 320–520 ms time window by means of two 3 × 2 ANOVAs (only on correct trials; Figure S2). The analysis yielded a significant main effect of the trial type in both frontal [*F*_(1.4, 11.4)_ = 24.75, *p* < 0.001, η^2^ = 0.593] and parietal [*F*_(1.4, 11.4)_ = 10.99, *p* = 0.001, η^2^ = 0.393] electrodes. The post hoc test showed significant differences in ERP amplitude between NonMon and Non-Target trials (*ps* < 0.001) and between NonMon and Target trials (*ps* < 0.003), but not between Non-Target and Target trials (*ps* > 0.093), in both scalp areas. These results demonstrated that the amplitude of the two examined ERP components did not differ between Target and Non-Target trials. A main effect of Domain emerged only in frontal sites, as in the previous block analyses [*F*_(1, 17)_ = 7.01, *p* = 0.017, η^2^ = 0.292]. No significant interactions emerged (*ps* > 0.290).

### ERP-fMRI results

**Figure 5:**
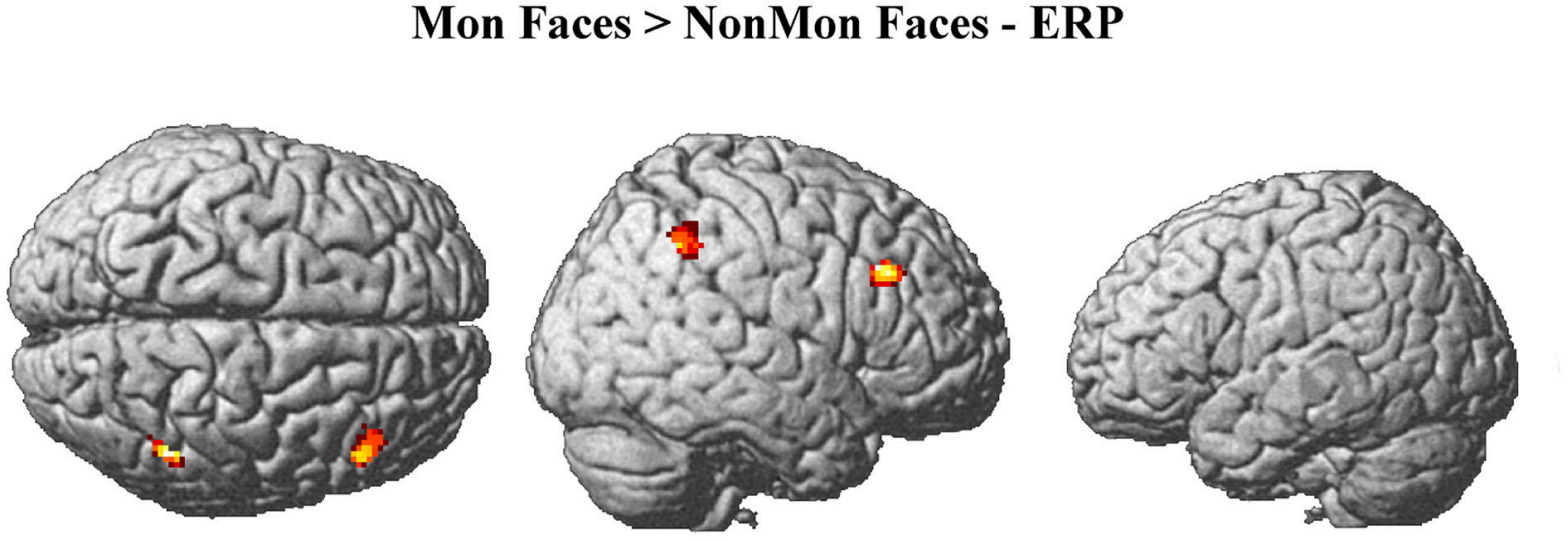
Brain activations modulated by the ERP amplitude over frontal electrodes (F1, F3, Fz, F2, F4) at *p* < 0.005 (voxel-level uncorrected) and *k* ≥ 52.

The monitoring-related ERP components which emerged in the conventional analyses were integrated with BOLD responses. Specifically, the mean amplitude of the ERPs over frontal electrodes (F1, F3, Fz, F2, and F4) in each block, from 320 to 520 ms, were entered in the first-level GLM as additional regressors (i.e., parametric modulator). Moreover, the mean ERP amplitude over parietal electrodes (PO3, POz, and PO4) in the same time-window was considered as a parametric modulator in a separate GLM (see the Statistical analysis section for details). No activations survived the *p* < 0.05 voxel-level FWE correction. Typically, EEG-BOLD coupling yields weak results since they derive from the residual effects after the mean BOLD responses are removed (Liu et al., 2016). Therefore we lowered the voxel-level significance threshold to a *p* < 0.005 uncorrected (Mulert et al., 2008). To control for multiple comparisons, the extent-threshold necessary to obtain a cluster-level FWE correction (*p* < 0.05) was derived from a Monte-Carlo simulation with 10,000 iterations (Slotnick and Schacter, 2004; https://www2.bc.edu/sd-slotnick/scripts.htm). Following this simulation, a cluster extent of a minimum of 52 contiguous voxels was considered. The activation of two right-lateralized clusters emerged when contrasting ERP regressors in Mon Faces vs. NonMon Faces (Table 3 and Figure 5). Namely, the activity of the right IPL, including the SMG, and of the right MFG were more activated in Mon compared to NonMon blocks and this activity was modulated by the frontal positivity potential. No clusters of voxels survived the chosen statistical threshold in the Tool domain and when considering parietal electrodes.

### Task difficulty

In order to check whether the increased sustained activity in the Monitoring condition was the expression of task-difficulty rather than monitoring processes *per se*, we correlated functional changes in brain activity with RTs (Burgess et al., 2003). Namely, the mean RTs for each block were entered into the design matrix as parametric modulator instead of the ERPs. The same significance criterion was adopted (*p* < 0.005 uncorrected, cluster extent threshold = 52 voxels). At the second level analysis, a 2 × 2 full-factorial ANOVA model was built. The Mon > Ong contrast revealed that only one cluster was modulated by RTs, namely the posterior portion of the left inferior and middle temporal lobe (*k* = 146, peak coordinates = −48, −58, −10; −50, −60, 0; −42, −58, 2). This area fell outside the mask created by the original contrast (Mon > Ong, fMRI results), meaning that task difficulty cannot account for the increased brain response in the Monitoring condition. The involvement of the posterior temporal lobe was likely linked to the sematic retrieval process that supported the categorization tasks, especially for tools (Whatmough et al., 2002; Kellenbach et al., 2003; Whitney et al., 2012).

## Discussion

The goal of the present study was twofold: (1) to investigate the neuroanatomical correlates of sustained and transient control processes mediating monitoring, and (2) to elucidate whether these brain structures are influenced by the nature of the to-be-processed material or are domain-independent. We referred to monitoring as the process of checking the environment over time for detecting the occurrence of target events (event monitoring). To address the first study goal, the EEG-fMRI coregistration technique was applied to a blocked experimental design, in which blocks requiring monitoring were contrasted to blocks not requiring monitoring. This approach enabled us to simultaneously capture both tonic (block-related fMRI) and phasic (event-related potentials) brain responses associated with the monitoring requirements. Conventional fMRI and ERP analyses were performed along with fMRI-ERP integration analyses (Debener et al., 2006; Mulert et al., 2008; Eichele et al., 2009). Namely, the block-by-block variations of ERPs were coupled with the corresponding block-by-block BOLD signal. To address the second goal, a within-subject design was used, in which participants were asked to monitor either faces or tools, in separate blocks. Task-domain specificities of monitoring were examined by comparing brain activity between the two types of blocks. These two stimulus materials were chosen because their processing is known to engage differently lateralized neural substrates.

The conventional fMRI results showed a set of fronto-parietal regions in Mon compared to NonMon blocks commonly active in both domains, confirming our expectations that event monitoring has a domain-general nature. The electrophysiological results revealed that the transient component of monitoring, elicited by the onset of each event (i.e., the stimulus-evoked or reactive control processes) were represented by ERP amplitude modulations from 320 to 520 ms after stimulus onset. The frontal ERP modulation correlated with the BOLD signal in the right IPL and right MFG for the face domain. No significant activations were detected for tool monitoring. A more detailed discussion of the functional significance of the results is provided below.

### Monitoring vs. non-monitoring

The fMRI findings confirmed previous evidence that points to the involvement of a fronto-parietal cortical network in monitoring the occurrence of target events over time (Reynolds et al., 2009; Vallesi et al., 2009; Vallesi and Crescentini, 2011; McDaniel et al., 2013; Benn et al., 2014; Vallesi, 2014). This network included the pre-SMA, the IFG, and lateral portions of the MFG at the frontal level, and the angular gyrus, the supramarginal gyrus and portions of the SPL at the parietal level. The activation of these areas reflected the action of tonic processes, which span multiple trials, across ITIs, and are not just instantiated at trial onsets. The conjunction analysis extended this evidence by showing that a similar bilateral set of fronto-parietal regions was recruited in Mon blocks compared to NonMon blocks in both stimulus domains. These results revealed the domain-general nature of the sustained control processes involved in monitoring and converged with the study of Benn et al. (2014) that found that long-lasting monitoring was associated with the bilateral activity of a fronto-parietal network of areas for both numerical and visuo-spatial domains.

The bilateral involvement of the fronto-parietal areas suggested that these processes required the cooperation of multiple cortical areas between the two hemispheres. Furthermore, the fact that the identified areas belong to distinct functional resting networks led us to speculate that multiple control networks, not just the fronto-parietal one, were engaged for implementing the task. For example, the IPS and the IFG have been consistently found to represent central nodes of two attentional control systems, that are the “dorsal attention network” (DAN) and the “ventral attention network” (VAN; Corbetta and Shulman, 2002; Fox et al., 2005; Corbetta et al., 2008), respectively. The former system, IPS, SPL and frontal-eye-field, is hypothesized to play a key role in the top-down allocation of attention to goal-relevant and expected stimuli. The latter system, which is based on right-lateralized frontal areas comprising the temporo-parietal junction, extending into IPL, the IFG/MFG, the frontal operculum and the anterior insula, is thought to support stimulus-driven orienting of attention to relevant but unanticipated stimuli. Both systems integrate endogenous and exogenous signals, therefore there is not a strict dichotomy between them (Macaluso and Doricchi, 2013). The bilateral IPS activation in our study denoted the involvement of important nodes of the DAN, and specifically the top-down adjustment of the attentional focus according to the task goal (Langner and Eickhoff, 2013). On the other hand, the role of the right IFG might be attributed to the detection of relevant stimuli (Corbetta et al., 2008). Collectively, these two areas might have underlined the attention control requirements of our monitoring task.

The involvement of the bilateral PFC (including both dorsal and ventral regions) cannot be fully explained by attentional control processes. The sustained coactivation of these areas suggested the intervention of additional tonic/proactive control component, such as the maintenance of task goals (Shallice and Burgess, 1991; Bunge et al., 2001; Miller and Cohen, 2001; Sakai and Passingham, 2003). Indeed, goal representations, which contain information regarding task requirements (e.g., detect cooking tools), needed to be maintained in an active state throughout the block in order to boost the target checking process.

The activation of the pre-SMA cluster might have mediated the selection between task-set representations (Rushworth et al., 2002; Crone et al., 2006; Vallesi et al., 2015)^2^. Indeed, in the monitoring blocks two task-sets must be managed: the categorization task and the target detection task. Therefore, the pre-SMA involvement could be not strictly related to monitoring processes but also to dual-task requirements.

The IFG and the IPS bilaterally, together with the pre-SMA and the insula, has been found to be part of the working memory network (Wager and Smith, 2003 for meta-analyses; Owen et al., 2005; Rottschy et al., 2012). In the monitoring task, working memory might have likely played a role in updating task goals as new information becomes available. However, it could also be related to the updating of counting. Although participants were instructed not to use the counting strategy, we cannot exclude they were actually engaged in it.

Importantly, in order to identify which cortical areas among those active across the entire block mediated phasic monitoring processes, the individual block-by-block ERP mean amplitude was entered into the GLM as a parametric modulator. This analysis revealed that, for face monitoring, two right-lateralized regions, namely the IPL and the MFG, covaried with the frontal ERP modulations. According to our hypotheses, these areas are active throughout the block but in a more phasic manner relative to the rest of the fronto-parietal network. Their transient activation might subserve a more trial-related evaluation of the events. Notably, the anatomical location of these areas were very close to the IPL and the inferior-middle frontal gyrus reported in a previous fMRI study focusing on monitoring spatial trajectories (Vallesi and Crescentini, 2011). The authors demonstrated that these areas were maximally activated in predictable trajectories, suggesting that the more the external contingencies match expectations, the higher their activity is. Taking together the findings of the present study and those of this earlier one, we may infer that the activity of these areas is linked with phasic match/mismatch operations, which compare the actual stimulus to the expected one.

The modulations in ERP amplitude, evoked by the onset of each event, highlighted the role of phasic processes also involved in monitoring blocks. In particular, a frontal ERP component was modulated in terms of an increased positivity in Mon compared to NonMon blocks. This ERP result was consistent with the findings in a similar study on event monitoring (Capizzi et al., 2016) and in previous studies focused on strategic monitoring in prospective memory (Cona et al., 2012, 2015a). Such positive deflection has been interpreted as reflecting the general recruitment of greater resources devoted to maintain the focus of attention on the monitoring requirements.

When the individual block-by-block ERP mean amplitude was entered in fMRI analyses as parametric modulator in the GLM in the tool monitoring blocks, no activation cluster survived the statistical threshold. We may interpret this null result as reflecting the fact that in the tool domain a weaker transient activity, masked by a stronger sustained one, was engaged relative to faces. The stronger BOLD activity reported in monitoring tools compared to the faces, and the higher monitoring cost observed in response times, corroborate this tentative explanation. Further research needs to disambiguate this point.

The operational definition of monitoring adopted in the current study might be strongly related to the constructs of vigilant attention and prospective memory (e.g., Kliegel et al., 2008; Langner and Eickhoff, 2013). Unlike typical vigilant attention tasks, however, our monitoring manipulation implies a categorization task and not simple detection or discrimination operations. Furthermore, in order to counteract the right frontoparietal deactivation associated with the increased time spent performing low demanding attention task (Coull et al., 1998) and to discourage the automatic processing, the monitoring task was inserted in an ongoing task. Unlike prospective memory tasks, the goals to be fulfilled were explicitly updated at the beginning of each block so that memory demands were minimized. In addition, the frequency of the target occurrence was higher than in typical prospective memory paradigms and the block duration shorter. Yet, some processes are likely to be commonly engaged in these two types of tasks. Indeed, vigilant attention is mediated by a mainly right-lateralized network of cortical structures (including middle and ventrolateral PFC, intraparietal sulcus and insula) as well as subcortical ones. In prospective memory, the dorsal fronto-parietal network, including precuneus and DLPFC, is associated with a strategic monitoring process (Cona et al., 2015b for a meta-analysis). This process reflects the allocation of top-down attentional and memory processes required, respectively, to maintain the intention active in mind and to monitor the environment for detecting the PM cues (i.e., the stimuli associated with the intention to execute).

### Faces vs. tools

The fMRI results confirmed that the processing of the two stimulus materials chosen for investigating the supra-ordinate nature of monitoring was subserved by a differently lateralized brain network. As expected, face processing was subtended by mainly right-lateralized areas compared to tool processing, whereas tools were processed by more left-lateralized regions. Specifically, faces compared to tools elicited the activation of the portion of the right fusiform gyrus, corresponding to the fusiform face area, which is devoted to face detection and recognition (Kanwisher et al., 1997; Kanwisher and Yovel, 2006; Frässle et al., 2016). In addition, the contrast revealed a significant activation of the amygdala, bilaterally. The significantly higher activation of the amygdala in the presence of neutral faces is likely linked to the processing of socially-relevant features of faces, in this case race (Todorov and Engell, 2008; Todorov, 2012). Interestingly, it has been found that the amygdala is affected by habituation when ingroup but not outgroup faces are presented (Hart et al., 2000). This phenomenon is part of the “other-race” face effect (Platek and Krill, 2009), and it has been proposed to result from an implicit and automatic process due to cultural learning (Lieberman et al., 2005). Since in our study faces belonging to different races were displayed, the activation of the amygdala suggests an implicit categorization of race (but the risk of reverse inference should be acknowledged here). The conventional ERP results confirmed the presence of a well-known face-selective component in face processing, the N170 over occipito-temporal and temporo-parietal electrodes (Itier and Taylor, 2004; Rossion and Jacques, 2008; Nguyen and Cunnington, 2014).

As compared to faces, the tool processing engaged the activity of wider sets of clusters, more extended in the left hemisphere, which comprised the fusiform gyrus, the middle occipital gyrus and the middle temporal gyrus. This pattern of areas was coherent with the involvement of the lateral occipital complex (LOC), located on the lateral bank of the fusiform gyrus and extending ventrally and dorsally, which mediates object recognition processes (Grill-Spector and Malach, 2001; Perini et al., 2014). The data confirm that the object-sensitivity of the LOC is stronger in the left hemisphere when objects are compared to faces (Haist et al., 2010).

Additionally, the tool processing activated the left IPL, comprising the supramarginal gyrus and the IPS. These parietal regions, together with the middle temporal gyrus, store the representations of the movements associated with the object's use and are automatically engaged by viewing manipulable objects with a clear affordance (tools), independently of an overt motor output (Chao and Martin, 2000; Creem-Regehr and Lee, 2005). In particular, the left IPL stores hand-postures that can be used for planning object-directed actions (van Elk, 2014). Altogether, these findings suggest the involvement of both the ventral and the dorsal visual streams, deputed to the functional identification of an object, that is to its perceptual recognition and to its use, respectively (Goodale and Milner, 1992; Valyear and Culham, 2010).

The fact that monitoring in the tool blocks required longer response times is likely related to the specific task requirements. Indeed, the categorization of tools according to their use is more complex than the categorization of faces according to the race or age. The activation of the left inferior frontal gyrus in tools' processing might reflect the implicit use of some linguistic functions to support the categorization task (such as naming, Lupyan et al., 2012; Lupyan and Mirman, 2013). Furthermore, the activity of the left inferior frontal gyrus might be associated with the selection of semantic knowledges (Thompson-Schill et al., 1997).

The electrophysiological counterpart confirmed these inferences. Specifically, the ERP marker of tool processing was represented by a negative deflection over central and pericentral electrodes at 240–340 ms, significantly larger for tools than faces. This negativity reminds the anterior negativity found in a passive viewing task where tools were compared to graspable objects, whose role was attributed to the automatic access to motoric object properties (Proverbio et al., 2011).

Taken together, the fMRI and ERP results on face and tool processing were coherent with the previous literature in showing an opposite lateralization of face and object processing and validate our choice of stimuli.

## Conclusions

The present research provides direct neuroimaging evidence of the domain-general nature of the sustained monitoring processes by showing that the same set of fronto-parietal cortical areas were co-activated when monitoring the environment for different types of events. Remarkably, the integration of fMRI and ERP findings offered a novel window into the attempt to detect the neural bases of a transient monitoring component overlapping with the sustained one. Indeed, while the bilateral fronto-parietal activation subtend the monitoring processes in a sustained manner, only right-lateralized clusters, at least in the face domain, expressed the phasic/transient component of the monitoring process.

## Author contributions

VT and AV conceived the experimental design. VT and SF created the task and the stimuli set. VT, IM, and SF collected the data. VT performed the EEG analyses. IM provided engineering support to MR set-up and performed fMRI and EEG-fMRI analyses. FC provided medical supervision and constructive suggestions to the experimental setting. AV provided significant feedbacks and contribution throughout all study phases. VT and IM drafted the manuscript. All authors provided substantial and critical revisions of the manuscript, approved the final version of the manuscript and agree to be accountable for all aspects of the work.

### Conflict of interest statement

The authors declare that the research was conducted in the absence of any commercial or financial relationships that could be construed as a potential conflict of interest.

## Footnotes

^1^The Italian word specifically refers to tools usually found in a toolbox and used to fix or build things, not to general tools, such as scissors.

^2^We can rule out a role of pre-SMA in controlling or preparing motor outputs (Nachev et al., 2008) since the monitoring task does not require immediate extra motor responses.

## Supplementary material

Figure S1 In **(A)**, the mean Global Field Power (GFP) between Faces and Tools is contrasted in Non-Monitoring (left) and Monitoring (right) blocks. In **(B)**, the mean GFP between Non-Monitoring and Monitoring blocks is contrasted in Face (left) and Tool (right) blocks. The gray frames mark the time-windows in which paired *t*-test denoted significant differences (*p* < 0.05).

Figure S2 Grand-average waveforms of faces-locked ERPs (left) and tools-locked ERPs (right) in Non-Monitoring blocks compared to NonTarget and Target stimuli in Monitoring blocks. **(A)** depicts frontal electrodes, parietal **(B)** depicts parietal electrodes.
