# Supplementary figures and images for: The Neural Bases of Event Monitoring across Domains: a Simultaneous ERP-fMRI Study
Source: Front Hum Neurosci. 2017 Jul 21;11:376. doi: 10.3389/fnhum.2017.00376 (PMC5519569; doi:10.3389/fnhum.2017.00376)

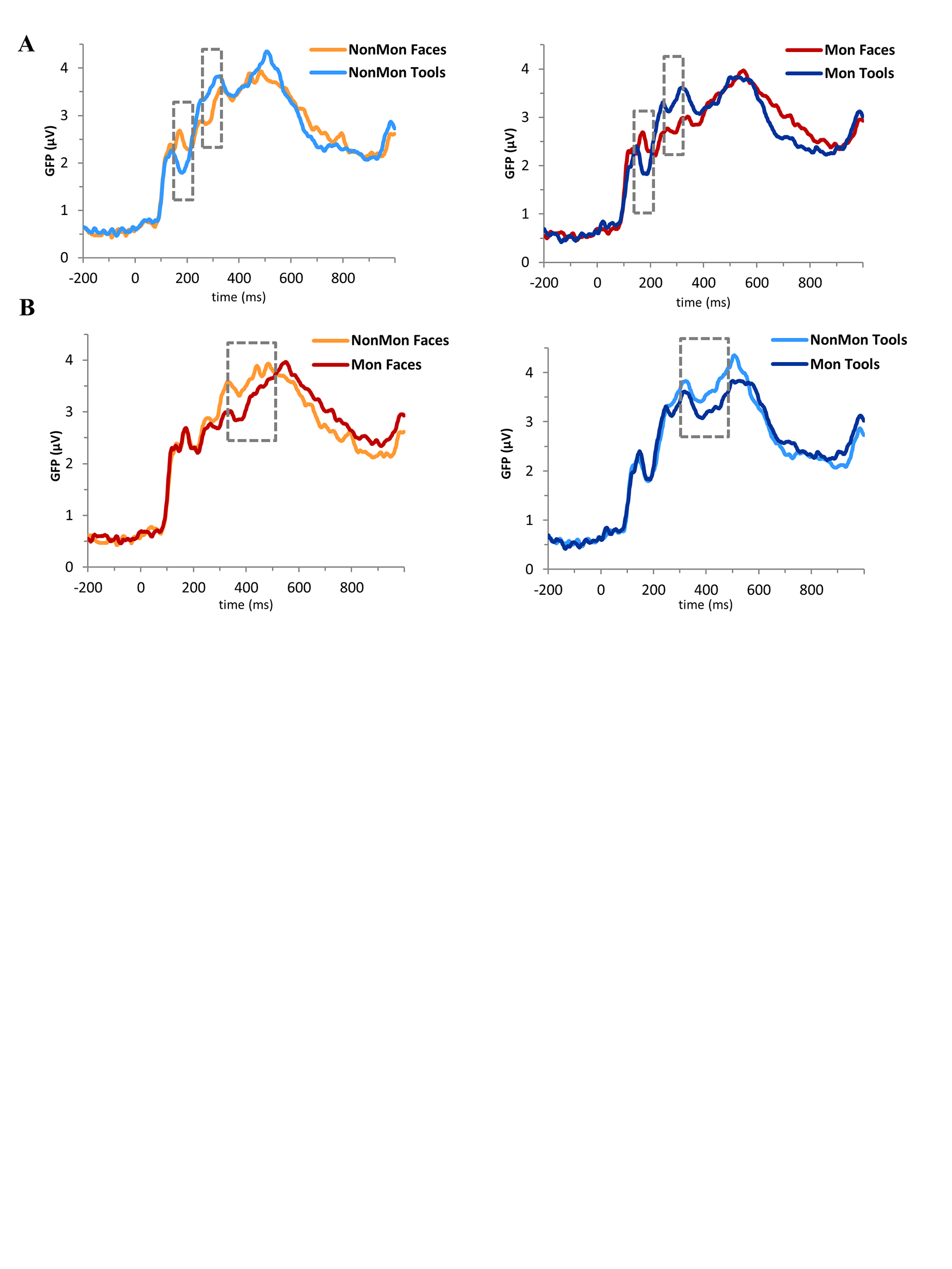

Supplement: Figure S1 — In (A), the mean Global Field Power (GFP) between Faces and Tools is contrasted in Non-Monitoring (left) and Monitoring (right) blocks. In (B), the mean GFP between Non-Monitoring and Monitoring blocks is contrasted in Face (left) and Tool (right) blocks. The gray frames mark the time-windows in which paired t-test denoted significant differences (p < 0.05). [file Image1.TIF]

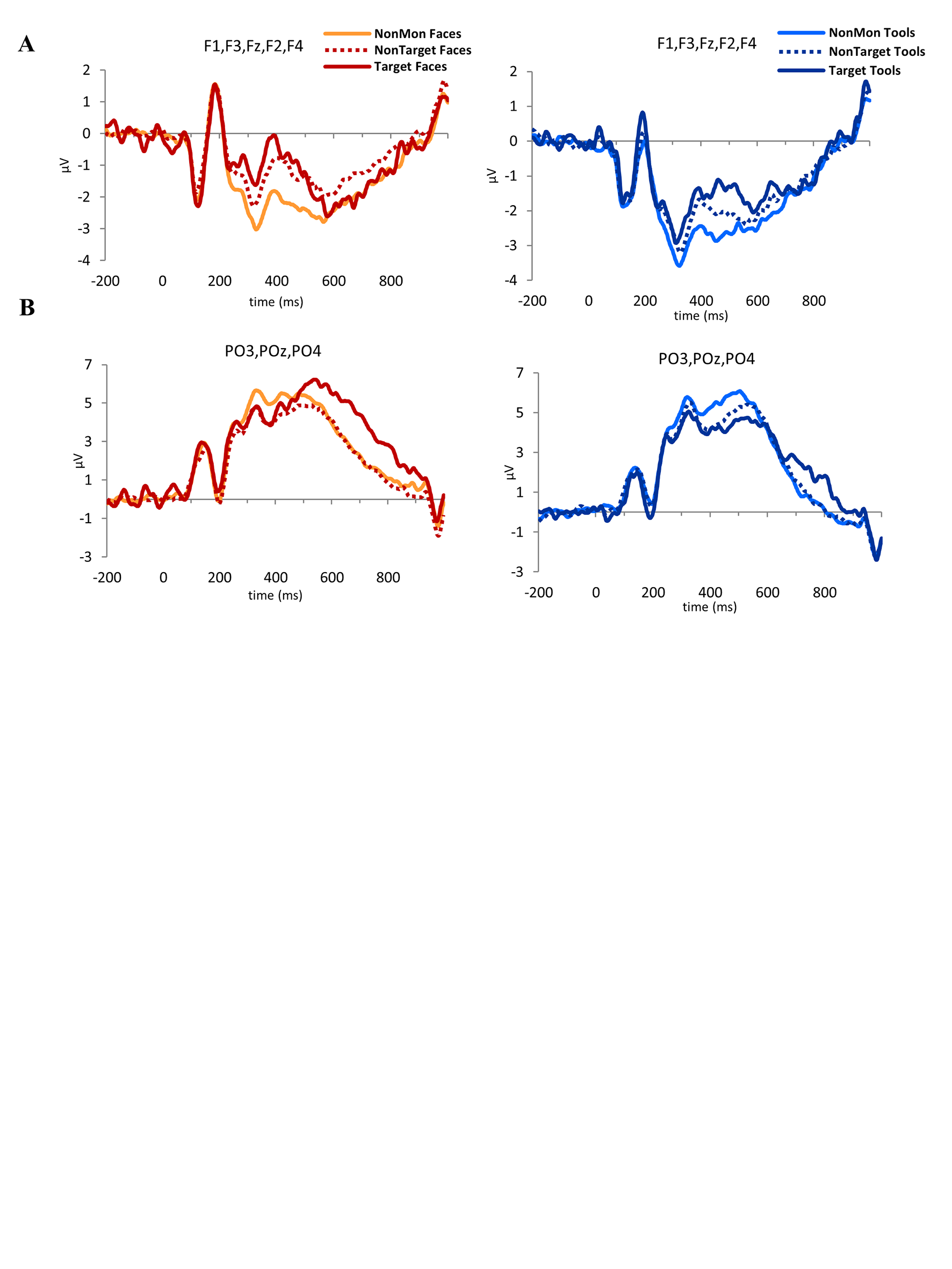

Supplement: Figure S2 — Grand-average waveforms of faces-locked ERPs (left) and tools-locked ERPs (right) in Non-Monitoring blocks compared to NonTarget and Target stimuli in Monitoring blocks. (A) depicts frontal electrodes, parietal (B) depicts parietal electrodes. [file Image2.TIF]
